# Thin and Scalable Hybrid Emission Filter via Plasma Etching for Low-Invasive Fluorescence Detection

**DOI:** 10.3390/s23073695

**Published:** 2023-04-03

**Authors:** Erus Rustami, Kiyotaka Sasagawa, Kenji Sugie, Yasumi Ohta, Hironari Takehara, Makito Haruta, Hiroyuki Tashiro, Jun Ohta

**Affiliations:** 1 Division of Materials Science, Graduate School of Science and Technology, Nara Institute of Science and Technology, 8916-5 Takayama, Ikoma 630-0192, Japan; 2 Department of Physics, Faculty of Mathematics and Natural Sciences, IPB University (Bogor), Kampus IPB Dramaga, Bogor 16680, West Java, Indonesia; 3 Department of Health Sciences, Faculty of Medical Sciences, Kyushu University, 3-1-1, Maidashi, Higashi-ku, Fukuoka 812-8582, Japan

**Keywords:** implantable device, fluorescent imaging, contact imaging, image sensor, emission filter, optical filter fabrication

## Abstract

Hybrid emission filters, comprising an interference filter and an absorption filter, exhibit high excitation light rejection performance and can act as lensless fluorescent devices. However, it has been challenging to produce them in large batches over a large area. In this study, we propose and demonstrate a method for transferring a Si substrate, on which the hybrid filter is deposited, onto an image sensor by attaching it to the sensor and removing the substrate via plasma etching. Through this method, we can transfer uniform filters onto fine micrometer-sized needle devices and millimeter-sized multisensor chips. Optical evaluation reveals that the hybrid filter emits light in the 500 to 560 nm range, close to the emission region of green fluorescent protein (GFP). Furthermore, by observing the fluorescence emission from the microbeads, a spatial resolution of 12.11 μm is calculated. In vitro experiments confirm that the fabricated device is able to discriminate GFP emission patterns from brain slices.

## 1. Introduction

Fluorescence microimaging, which involves a micro-sized CMOS image sensor to detect fluorescence emission, is essential in the biomedical field, especially for research with limited space and weight restrictions. Due to its lensless structure, wherein an image sensor detects an object without a lens, several hundred-micrometer devices can constitute the image sensor [1,2]. One of the primary applications of this sensor is visualizing and quantifying brain cell and tissue activity in freely moving animals [3,4,5,6,7,8]. Compared to other imaging modalities for behavioral experiments, such as miniature microscopy [9,10,11], a microimaging device enables multisite experiments by observing different brain areas simultaneously. This multisite observation is essential to decipher the principles underlying animal behavior in a specific brain region [2]. For deep brain imaging, miniature microscopes are equipped with an optical probe, such as an optical fiber or gradient-index (GRIN) lenses. Adding such components increases the overall weight and complexity of the microscope. Meanwhile, the contact imaging technique used in microimaging devices makes them flexible in determining the field of view, which is associated only with the pixel number.

In a lensless setup, an emission filter, which is an optical component that selectively transmits an emission, does not optimally reject the excitation light, as in the lens-based device [12]. Generally, the emission filter functions either through absorption or reflection. Polymer-based absorption filters have been widely used in various microimaging applications owing to their ease of handling and independent transmission spectra under various angles of incidence (AOIs) [13,14,15,16,17]. However, the autofluorescence characteristics of absorption filters do not facilitate a rejection level sufficient to produce a high-contrast image.

Meanwhile, an interference filter selectively reflects or transmits a particular spectrum, based on the interference. This reflection mechanism exhibits a high rejection ratio. In addition, the filter transmission spectrum, relying solely on the multilayered structural design, can be easily modified [13,18]. Therefore, the interference filter has been widely used in lens-based fluorescence devices [19,20]. In a lensless device, the interference filter suffers rejection due to the high AOI components generated via scattering, where the passband spectrum shifts toward the shorter-wavelength region [21].

Complementary interference and absorption filters in one hybrid filter structure provide a way to overcome the shortcomings of each type of filter. According to reports, this hybrid filter exhibited a higher rejection level than a single filter [22]. Moreover, by reducing the autofluorescence of the absorption filter, the hybrid filter increased the excitation rejection performance of a lensless device, making it comparable to a typical lens-based fluorescent microscope [23,24,25,26,27,28]. However, using this hybrid filter for microimaging devices requires thickness reduction to an acceptable invasiveness level. Other techniques, such as selectively transmitting light within a specific wavelength using semiconductor-based metasurfaces [29] and nanoplasmonics [30], have the potential for realizing ultra-small on-chip filter integration. However, using these techniques in a typical lensless imaging system requires a complex fabrication process.

In hybrid filters, the absorbing filter layer should be as thin as possible to minimize the degradation of the space fraction potential. For this reason, resin absorption filters with a high concentration of dyes have been used. However, the transfer method in [23,24,25] is an effective way to avoid this problem. However, it is challenging to handle ultrathin filters after they are peeled off. To address these issues, in our previous works, we transferred a thin hybrid filter onto a needle-type image sensor using a laser lift-off technique [31,32]. In this method, a high-energy laser generated heat to disassemble the interference filter from its substrate.

An interference filter and an absorption filter were formed on a quartz glass substrate, and then the image sensor was bonded to the substrate. The filters were then peeled off by irradiating a laser pulse of a 266 nm wavelength from the substrate side. Consequently, the needle sensor was able to identify the GFP emission from brain slices. However, in the case of a larger filter area, cracks emerged during laser ablation. In this case, as the separation area was considerably larger than that of a needle-type sensor, the heat from laser ablation might not be evenly distributed over the filter area, thereby leading to a mismatched thermal expansion between the filter and the glass substrate [33].

**Figure 1:**
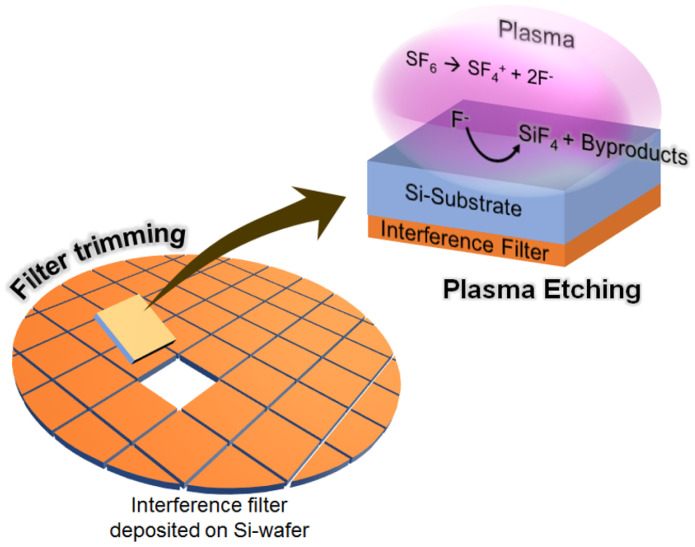
Schematic of the thin and scalable hybrid filter fabrication using the plasma etching technique.

This study proposes a novel method that uses plasma etching to integrate thin and scalable hybrid emission filters onto CMOS image sensors. A schematic of the thin hybrid filter fabrication using plasma etching is shown in Figure 1. Before initiating the etching process, the Si wafer, along with the interference filter, was deposited and trimmed to a handling size. Subsequently, in a plasma environment, the trimmed Si substrate chemically reacted with the etchant fluorine radicals (F${}^{-}$), a fragment of SF${}_{6}$ gas, yielding another gas and byproducts [34,35]. The entire Si substrate disappeared after a certain exposure time, leaving behind the free-standing filter attached to the image sensor. Since plasma etching relies solely on the chemical reaction, a large hybrid filter area can be fabricated by increasing the etching time.

## 2. Materials and Methods

### 2.1. CMOS Image Sensor

The CMOS sensors used in this report were designed in our lab and fabricated using the standard CMOS fabrication process (0.35-μm two-poly four-metal CMOS; Austria Microsystems). Among the various imaging devices available in our laboratory, a needle-type sensor, similar to that used in our previous imaging evaluation, was selected. This sensor has 40 × 400 pixels, a width of 500 μm, a length of 5100 μm, and a thickness of 150 μm. Detailed image sensor specifications are provided in Table 1. A dedicated electrical circuit board and graphical user interface (GUI) were built for processing an electrical signal from the image sensor to generate raw files, images, and movies. After data collection, a simple image processing program using ImageJ [36] with a plug-in (Fiji software) [37] and MATLAB (2018a, MathWorks, Natick, MA, USA) was used to determine the spatial resolution and merge the images for a larger FOV.

### 2.2. Filter Fabrication

**Figure 2:**
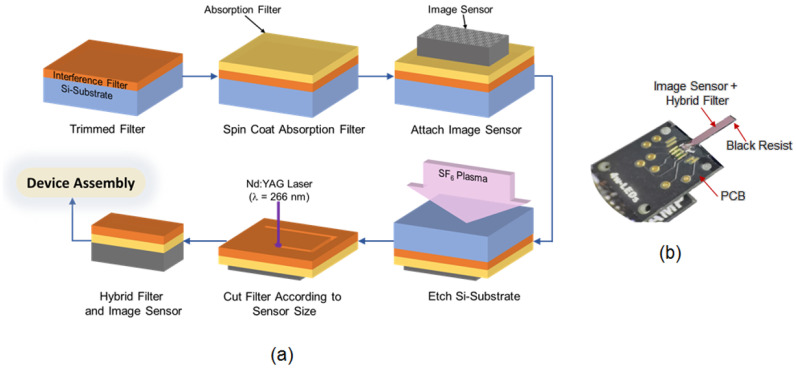
(**a**) Schematic of the hybrid filter fabrication using the plasma etching technique. (**b**) Photograph of the image sensor assembled with the designated printed circuit board.

First, the operational band of the interference filters was designed using the software designated in our lab. To ensure detection specificity, the filter transmission spectrum should be close to that of GFP emission, i.e., between 400 and 500 nm. Next, the interference filter was deposited on a 6-inch silicon wafer with a thickness of 600 μm instead of typical glass. The thin-film layers were deposited onto a silicon substrate using the ion-assisted deposition (IAD) method by a coating company (Tac Coat Co., Ltd., Nagano, Japan). The IAD method offers high throughput, comparable film quality, and lower operational costs compared to ion beam sputtering (IBS). This bulky 6-inch silicon wafer was manually trimmed to a size of 1 cm${}^{2}$ for ease of handling and then ultrasonically cleaned with acetone, isopropyl alcohol (IPA), and deionized (DI) water for 5 minutes after each stage. After the cleaning process, the filter entered the fabrication stage, which is described in the following steps (Figure 2).

1. *Absorption filter deposition*
  A yellow absorption filter (Valifast yellow 3150, Orient Chemical, Osaka, Japan), cyclopentanone (Wako, Japan), and NOA63 (Norland Product, Jamesburg, NJ, USA) were mixed at a weight ratio of 1:2:1, applied to the interference filter, and then spin-coated at 2000 rpm. Next, for pre-baking, the coated filter was photocured via ultraviolet (UV) irradiation (Omnicure 1000 spot-cure, EXFO, Tokyo, Japan) for 60 s. The subsequent curing step was performed by heating the sample at 100 °C for 15 min and hard-baking at 150 °C for 30 min.
2. *Image sensor attachment*
  A CMOS image sensor was attached to the yellow-coated filter using transparent epoxy resin (Z-1, Nissin resin, Tokyo, Japan), where the pixel area was directly in contact with the filter. Subsequently, the assembled materials were heated to 120 °C for 25 min and cured at 25 °C overnight before etching.
3. *Si-substrate etching*
  Following the reactive ion etching (RIE) machine sample preparation procedure (MUC-21 APS, Sumitomo Precision Product Co., Ltd., Hyogo, Japan), the assembled sample was attached to a micro-cover glass using polyvinyl alcohol (PVA). An additional PVA layer was applied to protect all the sensor sides from being attacked by the SF${}_{6}$ gas during the etching process. Next, the sample was assembled into a dedicated holder protected by Kapton tape and then inserted into the RIE chamber. Through the application of a continuous flow of SF${}_{6}$ gas at 130 sccm and a coil radio frequency (RF) generator at 812 W, a Si substrate with a thickness of 600 μm was etched in 90 min. This etching time was optimized to avoid the overexposure of the interference filter in the plasma environment. By raising the gas pressure and input RF power, the etching rate can be increased. However, we avoided overexposure in this investigation. Making the Si substrate thinner by lapping can reduce the etching time.
4. *Hybrid filter cutting*
  The etched filter was cut according to the CMOS sensor size using a 4th harmonic Nd:YAG laser (λ = 266 nm). This laser was also used to clean the chip pad area for electrical connectivity with an external circuit.
5. *Device Assembly*
  At this point, the assembly stage (including the printed circuit board (PCB) attachment, electrical wiring, and device protection) was similar to our previous device fabrication stage [31]. Figure 2b shows the final imaging device connected to the external PCB after the assembly stage. The thickness of the filter and bonding layer was approximately 20 μm, which was more than that in our previous work [31].

## 3. Results

### 3.1. Surface Filter Characterization

**Figure 3:**
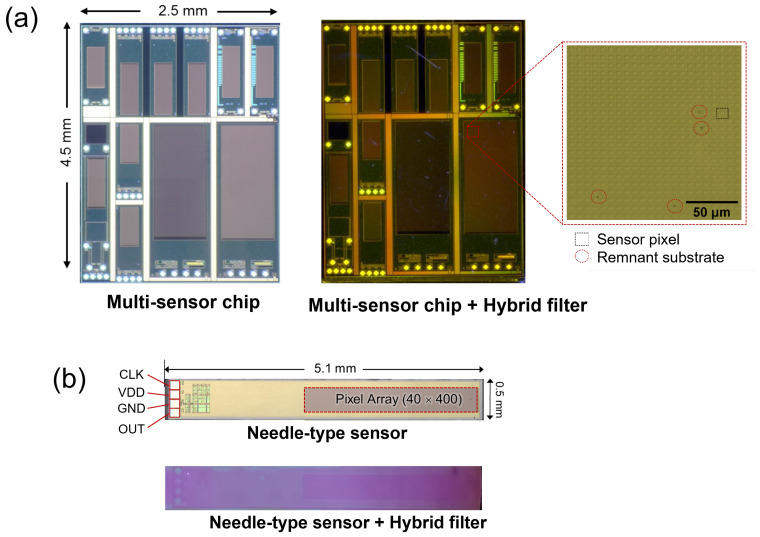
Photographs of the image sensor before and after filter deposition. (**a**) After etching, the multisensor area in the chip is flawlessly covered by a hybrid filter. The enlarged image shows the Si remaining on the filter post-etching (black dots). (**b**) Uniform hybrid filter covering the entire area of the needle-type sensor after etching.

Before optical characterization, a typical light and digital microscope (VHX-2000, Keyence, Osaka, Japan) were utilized to visualize the filter surface and check the remaining Si substrate after etching. This imaging approach displayed the remaining Si substrate compared with the CMOS pixel size. Two image sensors were integrated with the interference filters: unseparated multisensor and single needle-type image sensors. The multisensor chip, containing 11 different image sensors, was selected owing to its large dimensions, i.e., 11.25 mm${}^{2}$ (Figure 3a). Because our laboratory’s image sensors were designed for implantable applications, determining a single image sensor size equivalent to that of the multisensor chip size was challenging. For this multisensor chip, a long-pass interference filter was assembled not only for simpler preparation compared with that of a hybrid filter, which contains an absorption filter layer, but also for its wide transmission band that allows the light from the microscope to reach the pixel.

Furthermore, a needle-type image sensor, which was smaller than the multichip sensor, was selected for examining the hybrid filter. This image sensor was identical to the one used in the laser lift-off method [31]. Figure 3b shows a uniformly coated hybrid filter surface over the image sensor. This level of uniformity could not be achieved using the laser lift-off method, which inherently produces a laser ablation footprint [33]. Referring to the enlarged image of the long-pass filter, the etched filter contained no significant remnants of Si that could affect the image sensor’s detection sensitivity.

### 3.2. Filter Transmission Spectrum Profile

**Figure 4:**
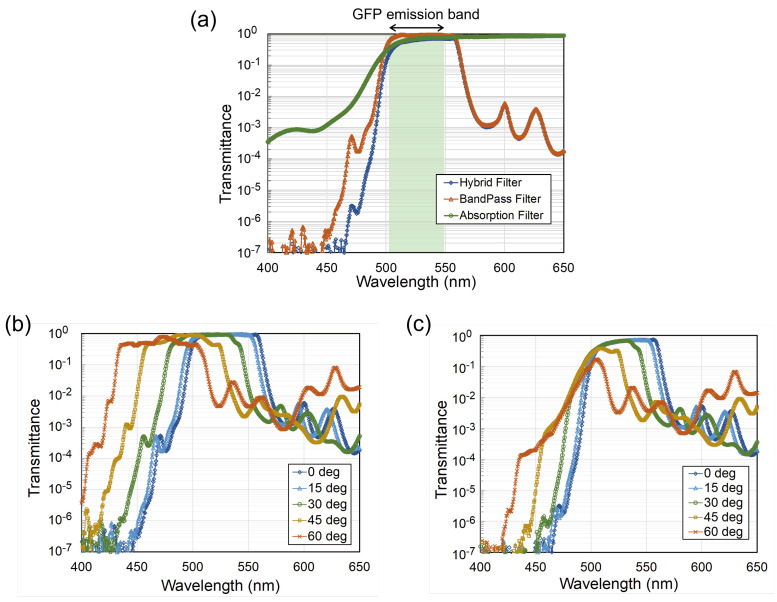
(**a**) Filter transmission spectra compared with the green fluorescence protein (GFP) emission on a logarithmic scale. (**b**) Transmission spectra of the interference filter with the various angles of incidence. (**c**) Transmission spectra of the hybrid filter with the various angles of incidence.

Regarding the filter performance, the transmission spectrum and rejection level of the hybrid filters, as well as each filter component, were examined. For the optical characterization, the interference filter was deposited on glass to ensure transmission. Although the conditions were slightly different, the filter spectrum characteristic was almost identical to the transferred one because the etching process did not change the interference filter structure. The filter transmissions were measured using a spectrometer (V-760, Jasco, Hyogo, Japan) with a normal incident angle. Figure 4a shows the filter transmittance of the bandpass, yellow absorption, and hybrid filters on a logarithmic scale compared with the normalized intensity of the GFP fluorescence emission. Similar to other interference filters, the bandpass filter had a steep and high rejection ratio reaching $10^{7}:1$ at around 430 nm. Owing to this feature, the bandpass filter achieved a high rejection ratio of the hybrid filter. Moreover, the bandpass filter had a narrow spectrum; it transmitted light from 500 to 560 nm, close to the GFP region. Assigning the bandpass filter in the outermost region reflected the blue excitation light and undesirable red fluorescence emission from biological substances, thereby ensuring that the green light passed through the image sensor.

The yellow absorption filter, deposited underneath the bandpass filter, acted as a shallow-steep long-pass filter, essentially absorbing the blue excitation light. Similar to other dye-based absorption filters, this yellow filter had a rejection ratio lower than that of the interference filter, being less than four orders of magnitude. However, despite the low rejection ratio, the yellow filter contributed to the rejection of tilted light components with high AOIs.

To ascertain the transmittance parameter, the ability of the hybrid filter to maintain its transmission spectrum was examined under various incident angles. As shown in Figure 4b, the bandpass filter transmission spectrum shifted toward a shorter region with the increasing AOI. At the most oblique position (60°), the bandpass filter shifted its transmission band to a shorter region from 430 to 510 nm. Within this new transmission spectrum, the blue excitation light specific to the GFP ($\lambda_{ex}$ = 473 nm) passed through the bandpass filter, thereby reducing the contrast image.

Conversely, the hybrid filter was able to maintain its blue light rejection for every AOI. The yellow absorption filter attenuated this blue shift from the bandpass filter and conserved the shorter region of the hybrid filter spectrum. As shown in Figure 4c, for the high AOI (30° to 60°), the hybrid filter spectrum, particularly in a shorter wavelength region, followed the yellow filter spectrum. Moreover, the blue shift occurred only in the longer wavelength region where the band-edge was solely associated with the bandpass filter. Consequently, this single-side shifting phenomenon pushed the hybrid filter’s transmission closer to the GFP region, thus increasing the detection specificity rather than jeopardizing it.

### 3.3. Spatial Resolution Examination

Spatial resolution is the ability to resolve ultra-small objects from the FOV. This resolvability can be examined using the emission from a micrometer-sized fluorescent bead, particularly for the lensless device. When two closely located objects are resolvable, their emission intensity can be distinguished from each other, resulting in two peaks instead of one. Moreover, this emission intensity was plotted for better quantization to obtain a full-width half-maximum (FWHM), which represents the shortest distance at which two overlapping light intensities are resolvable.

The fluorescence emission was measured from yellow fluorescent beads (FP-3052-2, Spherotech, IL, USA). With a diameter range of 2.5–4.5 μm, which was smaller than the dimension of the pixel sensor, the beads could be considered as the light source points. In addition, according to the specifications provided by the manufacturer, the excitation and emission peaks were 470 and 485 nm, respectively, similar to the GFP spectrum characteristics. Herein, blue light from a tabletop fluorescence microscope (BX51W1, Olympus, Japan) with a 10× and 0.1 NA objective lens was used for the excitation light. The beads on the surface filter were imaged under dry conditions to avoid spatial degradation.

**Figure 5:**
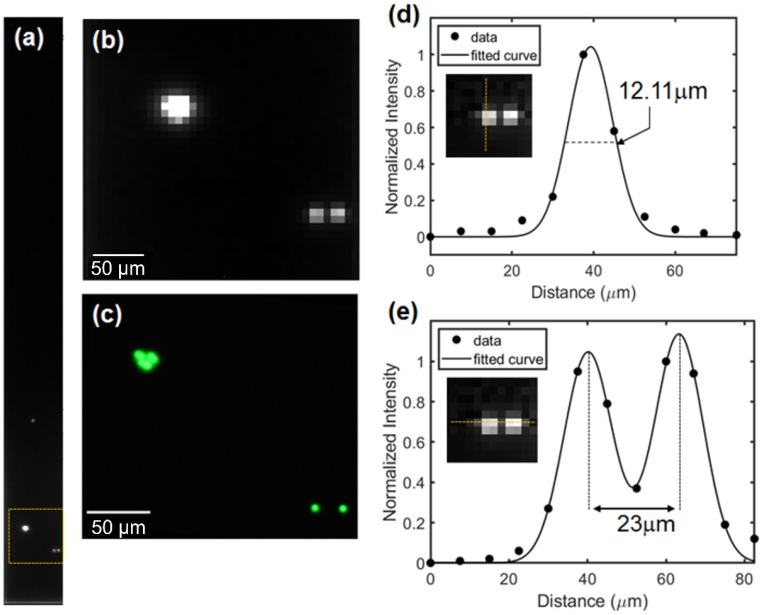
(**a**) Photograph of the microbead emission captured by the needle sensor. (**b**) Enlarged image of the ROI, denoted with a yellow square on (**a**) the merged beads in the upper left corner and the separated beads in the lower right corner; this image is compared with the microscope image (**c**). (**d**) Single-microbead emission intensity in the vertical direction, plotted and fitted with the Gaussian distribution. The FWHM is 12.11 μm. (**e**) Two close microbeads can be distinguished, as the distance between them is almost twice the FWHM.

Due to the narrow beam width of the irradiated light, not all the beads received the excitation light and emitted fluorescence. As shown in Figure 5a, only the lower image contained the bead emission, whereas the remaining areas were almost uniformly dark. This contrasting image indicated that the hybrid filter effectively rejected the excitation light. Further examination was conducted to determine the spatial resolution by selecting a region of interest (ROI) indicated by the dotted-yellow square. The enlarged ROI image showed merged beads in the upper left corner and two separated beads in the lower right corner (Figure 5b). These separated beads were then compared to the fluorescent image from the tabletop fluorescence microscope (Figure 5c).

To avoid interference from neighboring emissions, the single bead intensity was plotted on a vertical axis, denoted by the yellow line in the inset image of Figure 5d. From the intensity plot, which was fitted with a Gaussian profile, the FWHM was calculated as 12.11 μm. With this FWHM value, the two beads separated by approximately 23 μm were easily distinguished (Figure 5e). When the distance between neighboring beads was smaller than the FWHM, each intensity was not resolved and was considered a single emission for the merged beads in the upper left corner.

The spatial resolution of lensless devices degraded with the increasing distance from the object to the image sensor, based on the filter thickness. Thus, achieving a high rejection level while maintaining the filter thickness within an acceptable range was one of the main challenges in the lensless fluorescence setup. The new hybrid filter structure comprised a bandpass filter and a single yellow absorption filter. The long-pass filter of the bandpass structure limited the incident angle of the green fluorescence and reduced the blur in the fluorescent image, whereas the yellow absorption reduced the blue shift of the bandpass filter at a high angle of incidence. Employing a single absorption layer was beneficial for preserving the spatial resolution. Another fluorescence filtering method that reduces the distance, i.e., a filterless imaging device using a Bayesian filter, could increase the spatial resolution. However, the low rejection level of this internal filter mechanism should also be considered.

### 3.4. In Vitro Imaging

Subsequently, the GFP emissions were captured from 100-μm thick brain slices extracted from an adult gene-modified mouse. The brain slice was directly placed on the filter surface, and the blue excitation light from the fluorescence microscope illuminated the brain slice at a normal AOI. To avoid the overexposed illumination of brain slices, the excitation light intensity was adjusted by inserting additional filters and controlling the light aperture. All sample preparation procedures were performed following the guidelines of the Nara Institute of Science and Technology.

**Figure 6:**
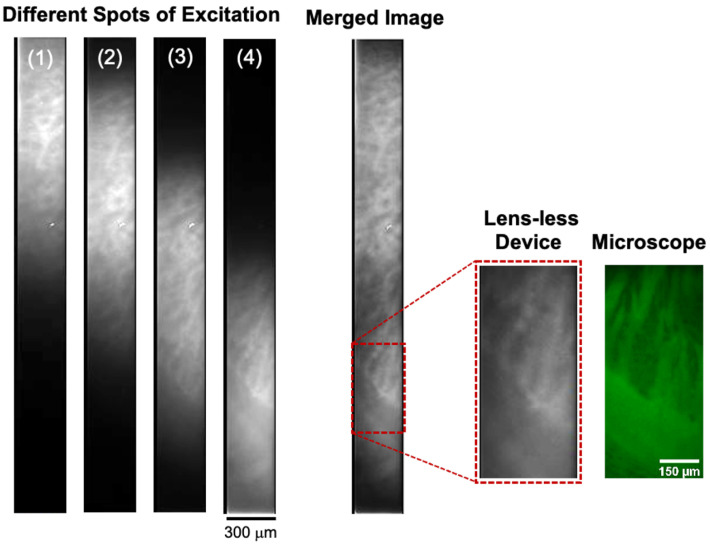
Fluorescent image from a brain slice captured by the needle sensor integrated with the hybrid filter. Owing to the narrow excitation light, four different irradiation spots (1–4) are required to excite all the sensor imaging areas. These images are merged for a large FOV by simple image processing. An ROI of the merged image, denoted by the dashed square, is enlarged and compared with the image obtained by the fluorescence microscope.

Exciting brain slices using a microscope’s light has several benefits owing to its narrow spectrum; nonetheless, as in the beads experiment, the beams were insufficient to cover all of the imaging areas. Figure 6 shows that to cover all of the needle-sensor imaging areas, at least four irradiation spots were required (images 1–4). Evidently, each image exhibited different brightness patterns associated with the GFP emission from neuronal cells. The darker area represented the absence of emission. When the excitation light shifted to the next spot, a new bright pattern emerged, and some previous bright patterns remained unchanged. These images were merged to obtain a large FOV, and no extra image processing was implemented. The merged image showed a moderate-contrast GFP emission over the needle-sensor imaging area. An observable white dot in the center imaging area may have resulted from the impurities on the filter surface.

Subsequently, the pattern on the ROI (denoted by the red dashed rectangle in Figure 6) was compared with that from the fluorescence microscope. This ROI was selected as it contained two different types of neural cells: a fine structure in the lower part and a large structure in the upper part of the image. Notably, the fabricated devices possessed a relatively similar profile of GFP emission yet with a lower contrast than that of the microscope image because of the spatial resolution limitation. Meanwhile, a slightly different GFP emission pattern between the needle sensor and microscope emerged from the mismatched observation areas. However, this pattern difference did not affect the needle sensor performance in identifying the GFP emissions from brain slices.

## 4. Discussion

The critical issue in thin hybrid filter fabrication is transferring a fragile interference filter, which is typically already deposited on a rigid and stable substrate, onto an image sensor. Herein, a method for transferring a thin hybrid filter was proposed by removing the entire Si substrate, allowing for successful integration not only in a finite needle-type sensor, as achieved by the laser lift-off method [24], but also in a multichip sensor that was five times larger than the needle-type sensor. Although further research on the assembly process is required, this large filter area opens the possibility of realizing multi-device fabrication. Various fluorescence microimaging devices with a uniform hybrid filter can be fabricated through a single etching process. Another unexplored area of Si-based interference filters is fabricating a particular microstructure on the filter, such as a waveguide, through a full DRIE process with an etching and passivation process.

The etching process, which relies on a chemical reaction, yielded a uniform and flawless filter. However, some particles remained unetched on the filter surface, possibly due to impurities on the surface before etching or a flawed etching process. As SF${}_{6}$ gas specifically reacts with Si and does not etch impurities, regardless of the etching time, these particles may remain. Although they were not sufficiently large to block incoming light and degrade pixel detection, extra cleaning processes, such as dry cleaning, can be utilized to reduce impurities before commencing the etching process.

The hybrid filter exhibited a bandpass transmission profile close to the GFP emission and suppressed the blue excitation light and autofluorescence in the red channel. As the passband blue shift only emerged in the longer region, the excitation rejection level remained almost unchanged for all the AOIs. Moreover, although the peak discrepancy between the filter transmission and the GFP emission did not affect the detection specificity, selecting the absorption filter with a steep-slope transmission may shift the filter transmission peak toward a shorter wavelength region, thus increasing the detection sensitivity.

Next, we examined the fluorescence imaging performance of the fabricated device. The spatial resolution, calculated from a single microbead emission, was 12.11 μm, approximately three times larger than that of the original beads. This degradation in spatial resolution was primarily caused by the increasing distance between the beads and the pixel sensor due to the hybrid filter. Additionally, since our imaging device did not employ extra functional components or specific post-image processing to enhance spatial resolution, the achieved spatial resolution could not be lower than the pixel sensor size, which was 7.5 μm. Nonetheless, the spatial resolution was higher than that of previous micro-image sensors with an absorption filter [6,14] and a hybrid filter [24]. From the perspective of the image sensor, selecting smaller pixel sizes can increase spatial resolution.

Meanwhile, because fluorescent light is spatially and temporally incoherent, holographic-based post-image processing cannot be used to achieve a superpixel resolution as in the Brightfield lensless device [38]. One possible way to overcome this is to obtain light-field information using angle-selective pixels [39,40]. Furthermore, despite the limited illumination requiring multiple, rather than single, irradiation spots to acquire the entire imaging area, the in vitro experiment confirmed that the fabricated device could identify the bright pattern of GFP emission from the brain slices. The low-contrast image obtained by the fabricated device resulted from the spatial resolution limitation. The contrast level can be improved for the neural cell larger than the achieved spatial resolution and in vivo experiment, which typically captures the GFP emission from living neuronal cells.

## 5. Conclusions

Herein, plasma etching was utilized to fabricate thin hybrid emission filters, comprising absorption and interference filters, for lensless microimaging devices. High-uniformity filters can be deposited on micrometer-sized needle-type and millimeter-sized multi-chip sensors, essentially relying on the chemical reaction between the fluorine etchant and Si wafer in a plasma environment. The hybrid filter transmission spectrum showed that the coalescence between each filter component ranged between 500 and 560 nm in a normal AOI, which is close to the GFP emission spectrum. Under a high AOI, the blue shift occurred only on the longer side of the hybrid filter passband, which did not jeopardize the excitation rejection performance. Subsequently, the fluorescence imaging performance was examined by detecting the emissions from microbeads and the GFP. Evidently, the needle-type sensor identified the microbead emission with a spatial resolution of 12.11 μm. Moreover, with this spatial resolution number, the GFP emission pattern was observed even for the brain slice interface area, which was noticeably distinguished.

## Figures and Tables

**Table 1:** Specifications of the needle-type CMOS image sensor.

Technology	0.35-μm two-poly four-metal standard CMOS
Operating voltage (V)	3.3
Pixel type	Three-transistor active pixel sensor
Pixel size (μm${}^{2}$)	7.5 × 7.5
Photodiode type	PSub–NWell
Pixel array	40 × 400
Color filter	None
Chip dimension (μm${}^{3}$)	500 (W) × 5100 (L) × 150 (H)

## Data Availability

Not applicable.
